# Queen Victorias Jubilee Institute for Nurses

**Published:** 1908-04-25

**Authors:** 


### Nursing Administration.

### QUEEN VICTORIA'S JUBILEE INSTITUTE FOR NURSES.

No one who has followed attentively the course pursued by the Jubilee Institute for Nurses can be surprised at the appeal for increased support which is now being made on its behalf. For many years after the founding of the Institute much misunderstanding existed with regard to its aims. People believed that the money had been subscribed for the purpose of paying the salaries of nurses to work in poor districts, and were often aggrieved when they discovered that the Queen's nurse had in effect to be paid at a somewhat higher rate than ordinary district nurses who had not received the same advantages in training. Grants in aid of local nursing associations were, it is true, frequently made in those early years, but the policy of devoting their main energies to the education and inspection of the nurses, leaving the work of support to local agencies, has been from the first consistently followed by the Council, and the result has abundantly justified them. The training required by the district nurse lies outside the ordinary nursing course, and it was evident that nurses at the completion of the orthodox training in general nursing, at the moment when funds were lowest, could not be expected, without grants in aid, to incur the expense of a supplementary course of instruction in the special problems of district work. Hence, before the era of the Jubilee Institute, the standard of training among district nurses was deplorably low, and they were too commonly recruited from the ranks of the partially trained.

True there were nurses who were drawn from the highest motives to dedicate their lives to work among the poor, and there were several District Nursing Associations in large towns which aimed at a high standard of work and training. But these exceptions only served to throw the general level of inefficiency into strong relief. The Jubilee Institute has from the first spent large sums every year in fitting women to undertake this work to the best advantage. In 1907 nearly £3,000 was spent in district training in England and Wales, and £589 in Ireland. During the year 168 nurses completed their training in district nursing in Great Britain, and 252 new nurses were placed on the roll of Queen's Nurses. It is to be regretted that, owing to the restless spirit which is one of the curious features of modern nursing, 213 nurses resigned during the year, of whom only 35 received the Leaving Badges to which nurses who have served at least six years are entitled. Yet this loss is to some extent compensated by the fact that 58 former Queen's nurses rejoined the Institute during the same period. It may be believed that, even in cases where Queen's nurses do not rejoin, they continue their work among the poor under other conditions, and that the training once bestowed remains a valuable asset for the nation. The Council of the Institute has always laid great stress on the importance of efficient inspection in the business of nursing the sick poor. The cost of inspection amounted in 1907 to over £2,000. There can be no doubt that the admirable system of inspection inaugurated by the Institute, and the successful choice of women to carry out this branch of the work, has had an enormous influence for good in raising the standard of life among the poor throughout the country. The inspection, carried out on good lines, is a continuation of the training already received. It is not only a guarantee to the subscribers that the work they have at heart is being efficiently carried through, it is the greatest possible encouragement and protection to the nurses employed in this arduous service, often in complete isolation from any who can appreciate their difficulties. In connection with this branch of the Council's work mention must be made of the wise policy which has taken under the ægis of the Institute the village nurses now doing good service in countless places where fully-trained nurses could neither be paid nor find sufficient occupation. It is certainly the easiest policy, and one which has been too generally followed in this country, to ignore altogether the secondary branches of the nursing service. The Jubilee Institute has, we think, acted courageously in grappling with the problems surrounding the partial training of nurses for small villages, and has already raised the standard, first by systematic inspection, secondly by lengthening the training given, and thirdly by confining the operations of the village nurse to places unsuitable to be the sphere of highly trained nurses. We look forward to a wide development of the work of the Institute in the direction of training midwives for country districts, not by the fallacious process of despatching them to institutions, but by placing them under qualified midwives in outdoor practice among the poor. Many new doors for the adequate training of midwives must be opened in the near future, and improved organisation would enable the Jubilee Institute to use their admirable staff of midwives in many parts of the kingdom for teaching purposes. For the institution-trained midwife, although she may be bound for a term of years by contract, rarely settles down to permanent work among the poor. Sooner or later she aims at the higher emoluments of the monthly nurse, and abandons her poorer clients. Hence it is to pupils trained among the poor, trained under the auspices of the Jubilee Institute, that the country must look in the approaching shortage of midwives. The advantage to the country of having a powerful organisation enlisted in the cause of nursing the sick poor is evident to all. The valuable results to which the Institute can point have been achieved through well-grounded confidence in the strength of local support for good objects, through the maintenance of a high standard of nursing, and through a generous consideration for the welfare of the nurses employed. Never was there any cause which could more securely rely on the generosity of the public.

